# Underdetermined Blind Source Separation of Audio Signals for Group Reared Pigs Based on Sparse Component Analysis

**DOI:** 10.3390/s24165173

**Published:** 2024-08-10

**Authors:** Weihao Pan, Jun Jiao, Xiaobo Zhou, Zhengrong Xu, Lichuan Gu, Cheng Zhu

**Affiliations:** College of Information and Artificial Intelligence, Anhui Agricultural University, Hefei 230036, China; panweihao17114212@stu.ahau.edu.cn (W.P.); jiaojun2000@ahau.edu.cn (J.J.);

**Keywords:** pig audio, signal sparsification, AP clustering, *l*_1_ norm, blind source separation

## Abstract

In order to solve the problem of difficult separation of audio signals collected in pig environments, this study proposes an underdetermined blind source separation (UBSS) method based on sparsification theory. The audio signals obtained by mixing the audio signals of pigs in different states with different coefficients are taken as observation signals, and the mixing matrix is first estimated from the observation signals using the improved AP clustering method based on the “two-step method” of sparse component analysis (SCA), and then the audio signals of pigs are reconstructed by L1-paradigm separation. Five different types of pig audio are selected for experiments to explore the effects of duration and mixing matrix on the blind source separation algorithm by controlling the audio duration and mixing matrix, respectively. With three source signals and two observed signals, the reconstructed signal metrics corresponding to different durations and different mixing matrices perform well. The similarity coefficient is above 0.8, the average recovered signal-to-noise ratio is above 8 dB, and the normalized mean square error is below 0.02. The experimental results show that different audio durations and different mixing matrices have certain effects on the UBSS algorithm, so the recording duration and the spatial location of the recording device need to be considered in practical applications. Compared with the classical UBSS algorithm, the proposed algorithm outperforms the classical blind source separation algorithm in estimating the mixing matrix and separating the mixed audio, which improves the reconstruction quality.

## 1. Introduction

Pig audio signals contain rich behavioral characteristics. Identifying pig audio signals makes it possible to analyze its state and then promote welfare-oriented breeding. At present, domestic and foreign scholars mainly focus on the endpoint detection of a single audio signal and the identification of a single audio signal in different states [1,2,3,4,5]. Pigs live in a group environment, so the collected audio signal is the aliasing signal sent by many pigs, which is not favorable for the direct analysis, feature extraction, and recognition of pig audio signals by modern information technology [6]. To separate a single source signal from the aliasing pig audio signal as much as possible and extract its effective audio features, blind source separation has become an effective solution.

Blind source separation (BSS) is to recover an unknown source signal from an aliasing signal captured by a microphone. In the past, BSS was mainly used in fields such as speech processing, biomedical signal processing, and mechanical failure [7,8,9]. Depending on whether the number of source signals is less than, equal to, or greater than the number of microphones, BSS can be divided into three cases: overdetermined, positive-determined, and underdetermined separations. Considering the large number of pigs on a farm and the small amount of signal acquisition equipment, this study focuses on the UBSS.

In recent years, scholars at home and abroad have adopted methods based on SCA [10,11] to solve the UBSS problem. For the SCA algorithm, the importance of the sparsity of the source signal is self-evident. Many extension algorithms in the time-frequency domain have been proposed to enhance and make full use of the signal sparsity. Zhen et al. [12] found and proved that the time-frequency point dominated by a single source point is related to a one-dimensional subspace; they used the hierarchical clustering algorithm to estimate the mixing matrix and solved a series of least squares problems to recover the source signal. Yuan Xie et al. [13] proposed an improved information theory criterion method to detect the number of source signals in the underdetermined case, estimated the mixing matrix using the fourth-order tensor blind identification method, and then reconstructed the source signals using a l_p_ norm diversity measure method, finally obtaining better separation results and higher running speed. Yu Hexin [14] proposed a time-frequency two-step method for the linear mixed UBSS problem of non-sparse signals and proved the effectiveness and accuracy of the algorithm through numerical experiments and analysis. Nie et al. [15] proposed a novel and efficient algorithm for solving row sparse principal component analysis problems without relying on any data structure assumptions. They transformed it into a solvable equivalent problem through coordinate descent and demonstrated its superiority through extensive experiments on real data sets. Fujimori et al. [16] proposed the Oracle inequality for the penalty principal component estimator for sparse principal component analysis in high-dimensional stable processes and elucidated the theoretical rate of parameter tuning for the penalty estimator. The performance and practicality of sparse principal component analysis were demonstrated through numerical simulations.

Currently, deep learning has good applications in various fields, and there are corresponding developments in the field of audio separation. Tzinis et al. [17] proposed a two-step training program for sound source separation using deep neural networks, which learned the optimal latent space and its inverse transform and trained the separation module in this space. The experiment showed that this method is superior to joint learning systems and has wide applicability. Xie et al. [18] constructed a BACPPNet model based on Deeplabv3plus utilizing a polarized self-attention mechanism for enhancement and achieved the separation of bird sounds in mixed audio, which is helpful for understanding wild bird sounds. Mancusi et al. [19] proposed a method for separating fish calls in ocean soundscapes through passive acoustic monitoring (PAM) by generating synthetic soundscapes to train two popular sound separation networks. The research results indicate that these separation models demonstrate their generalization ability on synthetic test sets and real data.

According to the above background information, the main subject of UBSS audio experiments is usually functional signals, but there is little research on the actual collected audio signals. The currently popular deep learning models require a large amount of data as support, and many fields cannot obtain such a large amount of data. In cases where the amount of data is very small, a new approach is needed. In view of this, based on the SCA “two-step method”, we propose an SCA-based UBSS method for audio signal separation in pig farming to solve the problem of underdetermined audio aliasing signals in pig farming environments.

## 2. Materials and Methods

### 2.1. Experimental Materials

In this study, NanoPc-T4 was used as the main controller, and iTalk-02 microphone, USB interface, and other hardware devices were connected to build an audio acquisition and transmission hardware system. The audio format was set to WAV, the sample size was 16 bits, and the sample rate was 44.1 kHz. The acquisition object was adult Landrace sows from Jinghuimeng Pig Farm in Mengcheng of Anhui Province, China. The audio signals were acquired in a quiet space and processed by Kalman filter to reduce noise. The experiment was completed in the hardware environment of 11th Gen Intel^®^ Core™ i5-1155G7 under Win10 operating system, and the software Matlab R2016a was used to create and implement the corresponding functions.

### 2.2. Overall Design of UBSS

**Figure 1:**
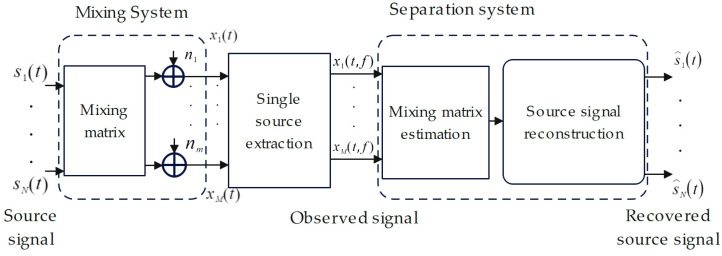
Linear transient mixed model.

UBSS used multiple sensors to measure and receive audio signals from multiple pig sources. When the number of the observed signals was less than the number of the source signals and the prior information was insufficient, all the source audio signals could still be accurately recovered from the multiple source audio signals. The framework of blind source separation system is shown in Figure 1. From the figure, it can be seen that the model mainly consists of a mixing system and a separation system. In the mixing system, the source signal $S(t)={\left[s_{1}(t),\;\ldots ,\;s_{N}(t)\right]}^{T}$ is mixed by an unknown mixing matrix ***A***, and noise is added to obtain an observed signal $X(t)={\left[x_{1}(t),\;\ldots ,\;x_{M}(t)\right]}^{T}$; the separation system takes the successful separation of the source signal as the execution objective. The closer the estimated value $\hat{S}(t)={\left[{\hat{s}}_{1}(t),\;\ldots ,\;{\hat{s}}_{N}(t)\right]}^{T}$ of the separated source signal is to the source signal S(t), the higher the operation quality of the separation system is.

### 2.3. Audio Mixed Signal Model

The mathematical expression of the linear transient mixed model of UBSS is as follows:

(1) X(t)=AS(t)+H(t)

where *t* is the time index; ***X***(*t*) and ***S***(*t*) are the mixed signal and sound source signal, respectively; and ***H***(*t*) denotes the noise. Assuming there are *M* observed signals and *N* sound source signals at the experimental site, and *M* < *N*, then $A={\left(a_{ij}\right)}_{M\times N}$ is the mixing matrix, $H(t)={\left[n_{1}(t),\;\ldots ,\;n_{M}(t)\right]}^{T}$, where $x_{m}(t)$ denotes the mixed signal received by the *m*-th receiver, and $s_{n}(t)$ denotes the *n*-th source signal. The influence of noise was not considered in this study, so ***H***(*t*) was set to 0.

### 2.4. Audio Signal Sparsification and Single Source Point Extraction

According to the analysis of mixed signal recoverability in reference [20], the more sparse the signal is in the time domain or transform domain, the higher the probability that each source signal can be correctly separated. Therefore, short-time Fourier transform (STFT) was applied to the pig mixed audio signal to enhance its sparsity [13]. Based on the short-term stationary property of non-stationary signals, there must be a single source point neighborhood with constant frequency and adjacent time, and the points in this neighborhood are all generated by the same source signal. If *N* source signals can be extracted from the mixed signal, the scatter plot composed of single source signals will be clearly clustered around *N* straight lines [21,22]. Then, using the clustering algorithm, it is able to estimate the mixing matrix ***A***. Equation (1) can be expanded into Equation (2), where each observed signal $x_{i}(t)=[x_{i}(1),x_{i}(2),\ldots ,x_{i}(T)]$, *T* is the number of sampling points, as follows:

(2) $$X(t)=\left[\begin{array}{c} x_{1}(t)\\ x_{2}(t)\\ \vdots \\ x_{M}(t) \end{array}\right]=\left[\begin{array}{cccc} a_{11} & a_{12} & \ldots & a_{1N}\\ a_{21} & a_{22} & \ldots & a_{1N}\\ \ldots & \ldots & \ldots & \ldots \\ a_{M1} & a_{M2} & \ldots & a_{MN} \end{array}\right]\left[\begin{array}{c} s_{1}(t)\\ s_{2}(t)\\ \vdots \\ s_{N}(t) \end{array}\right]=AS(t)$$

The above equation becomes X(t,f)=AS(t,f) after a short-time Fourier transform. In order to reduce the effect of low-energy points and the computational effort, the time-frequency coefficient X(t,f) of the observed signal matrix ***X*** is processed as follows at a certain time-frequency point $\left(t_{0},f_{0}\right)$, where e=0.05:

(3) $$X(t_{0},f_{0})=\left\{\begin{array}{cc} X(t_{0},f_{0}), & {\left\|X(t_{0},f_{0})\right\|}_{2}\geq e\\ 0, & {\left\|X(t_{0},f_{0})\right\|}_{2}<e \end{array}\right.$$

All time-frequency points are processed as described above to eliminate low-energy points. Assuming that at the time-frequency point $\left(t_{0},f_{0}\right)$, only the value of the source signal $s_{i}(t_{0},f_{0})$ is very large, while the values of the other source signals are zero or close to zero, Equation (2) can be approximated to the following:

(4) x1(t0,f0)x2(t0,f0)⋮xM(t0,f0)=a1ia2i⋮aMisi(t0,f0)⇔x1(t0,f0)x2(t0,f0)=Im(x1(t0,f0))Im(x2(t0,f0))=Re(x1(t0,f0))Re(x2(t0,f0))=a1isia2isi=a1ia2i

where $x_{i}(t_{0},f_{0})$ is the complex representation of the *i*-th observed signal in the time-frequency domain, and Re(⋅) and Im(⋅) denote the real and imaginary parts, respectively. From Equation (4), it can be known that at all the moments when the source signal $s_{i}(t_{0},f_{0})$ is nonzero, a straight line whose direction is the *i*-th column vector of the mixing matrix ***A*** will be determined, and the ratio of the real part to the imaginary part of the single source point is a constant value. Therefore, Equation (5) can be used as the criterion of a single source point:

(5) Im(x1(t0,f0))Re(x1(t0,f0))=Im(x2(t0,f0))Re(x2(t0,f0))

However, due to the influence of noise and calculation error, as well as a large number of low-energy points (points clustered near the zero point), the spatial distribution of the single source points extracted by Equation (5) will deviate from the column direction corresponding to the mixing matrix ***A***, resulting in a large error in the estimated mixing matrix. In view of this problem, we first used Equation (6) to relax the constraints to preliminarily screen single source points, where $\varepsilon_{1}$ is denoising threshold, $0<\varepsilon_{1}<1$:

(6) Im(x1(t0,f0))Re(x1(t0,f0))−Im(x2(t0,f0))Re(x2(t0,f0))<ε1

We take *G* neighboring time-frequency points as a neighborhood, and the domain for the *G* observed signal data points is called a subgroup. We calculate the variance of the observed signal within each subgroup to measure the degree of difference and set a threshold $\varepsilon_{2}$, $0.01<\varepsilon_{2}<0.5$. When the variance is less than $\varepsilon_{2}$, it will be retained, otherwise, it will be excluded. The following formulae are utilized to calculate whether the data points within each grouping satisfy the conditions separately. In the formula, $x_{i}(t_{m},f_{m})$ denotes the data within each subgroup of the *i*-th observation signal. ${\bar{x}}_{Gi}(t,f)$ denotes the vector consisting of the data mean of each subgroup of the *i*-th observation signal, and $\mathrm{var}[x_{Gi}](t,f)$ denotes the vector consisting of the data variance of each subgroup of the *i*-th observation signal.

(7) $${\bar{x}}_{Gi}(t,f)=\frac{1}{G}\sum_{m=1}^{G}x_{i}(t_{m},f_{m})$$

(8) $$\mathrm{var}[x_{Gi}](t,f)=\frac{1}{G-1}\sum_{m=1}^{G}{\left|x_{i}(t_{m},f_{m})-{\bar{x}}_{Gi}(t,f)\right|}^{2}$$

### 2.5. Calculation of the Mixing Matrix

To accurately calculate the mixing matrix ***A*** and the number of source signals, we adopted the clustering algorithm to cluster the data. Affinity Propagation (AP) clustering regards all sample points as potential cluster centers (exemplars) and selects the center points through loop iteration to obtain the optimal class representative cluster. Nevertheless, the clustering results are affected by the super-parametric damping coefficient, and the complexity of the algorithm is high. Thus, based on the singular value decomposition (SVD) method, we proposed an adaptive damping coefficient AP clustering algorithm, calculated the initial cluster center and the number of source signals, and then used K-means clustering algorithm to update the cluster center, and finally calculated the mixing matrix.

#### 2.5.1. Low-Rank Approximation of Matrices and Singular Value Decomposition (SVD)

When using AP clustering to construct the similarity matrix, the obtained dimension of the matrix is large, which requires a large amount of memory, and the calculation complexity is high. Therefore, the SVD was used to reduce the dimension, speed up the calculation of the algorithm, and reduce the complexity. The data of each time-frequency point after single source point detection is converted to a time-domain signal by an inverse short-time Fourier transform. Let the set of all mixed-signal data after single source point detection at this point be $X_{SVD}=\{x(1),x(2),\ldots ,x(Num)\}$, *Num* is the length of the signal sequence after single source point detection, then at this time the Hankel matrix is shown in the following equation. After the single source point detection processing in Section 2.4, the observed signal, which is reconstructed in the phase space, is truncated using a sliding window to construct the Hankel matrix $Ha\in R^{P\times Q}$. Assuming that the length of the signal sequence is *Num*, when *Num* is odd, *P* = (*Num* + 1)/2 and *Q* = (*Num* + 1)/2. When *Num* is even, *P* = *Num*/2 + 1 and *Q* = *Num*/2. The *r* in Equation (9) is the rank of ***Ha***.

(9) $$Ha=\left[\begin{array}{cccc} x(1) & x(2) & \ldots & x(Q)\\ x(2) & x(3) & \ldots & x(Q+1)\\ \vdots & \vdots & \vdots & \vdots \\ x(P) & x(P+1) & \ldots & x(Num) \end{array}\right]=U\Sigma V^{T}=U\left(\begin{array}{cc} \Sigma_{r} & 0\\ 0 & 0 \end{array}\right)V^{Τ}$$

where $U\in R^{P\times P}$ and $V\in R^{Q\times Q}$ are all orthogonal matrices, $\Sigma_{r}=diag(\sigma_{1},\sigma_{2},\ldots ,\sigma_{r})$ is a diagonal singular value matrix, the elements on its diagonal satisfy $\sigma_{1}\geq \sigma_{2}\geq \ldots \geq \sigma_{r}>0$, and *r* is the rank of the matrix ***Ha***, *r* ≤ min(*P*,*Q*). Take $U=(u_{1},\ldots ,u_{P})$ and $V=(v_{1},\ldots ,v_{Q})$, then $Ha=\sum_{i=1}^{r}\sigma_{i}u_{i}v_{i}$, in which $u_{i}$ and $v_{i}$ are separately called the left and right singular vectors of the *i*-th singular value $\sigma_{i}$ of ***Ha***.

#### 2.5.2. AP Clustering Algorithm and Mixing Matrix

The basic principle of the AP clustering algorithm is to calculate the similarity value between *L* data points [23], put the similarity value into the matrix $S^{\prime }$, select the reference threshold (the median of $S^{\prime }$), and set a maximum number of iterations; after the start of iteration, calculate the support matrix ***R*** value and the availability matrix ***W*** value, and then determine whether it is the cluster center according to the value of *R*(*k,k*) + *W*(*k,k*). When the number of iterations exceeds the maximum number of iterations or when the cluster center does not change for multiple iterations, the remaining points are divided into cluster centers according to the similarity [24,25]. The specific steps of the algorithm are as follows:

(1) Construct a similarity matrix. Let the set of all mixed-signal data at this point after SVD dimensionality reduction be $X_{AP}=\{x_{1},x_{2}\ldots ,x_{L}\}$ and *L* be the length of the data set. Using the similarity between Euclidean distance data points, as shown in Equation (10),

(10) $$s^{\prime }(i,k)=-{\left\|x_{i}-x_{k}\right\|}^{2}$$

where s’(i,k) denotes the similarity between data points *i* and *k*. The similarity between all data points forms a similarity matrix $S^{\prime }$, and the size of the similarity matrix will be L×L.

(2) Initialize the support matrix ***R*** and the availability matrix ***W*** as **0**.

(3) Update the support matrix ***R***. The support degree $r_{tb}(i,k)$ refers to the support degree of data point *i* for data point *k* as the cluster center. The *tb* represents the beginning value subscript at the current moment, *te* represents the final updated value subscript at the current moment, and *te*-1 represents the final updated value subscript at the previous moment. It can be calculated using Formula (11):

(11) $$r_{tb}(i,k)=\left\{\begin{array}{l} s’(i,k)-\underset{k\neq k’}{\max}\left\{w(i,k’)+s’(i,k’)\right\},i\neq k\\ -\underset{k\neq k’}{\max}\left\{w(k,k’)+s’(k,k’)\right\},i=k \end{array}\right.$$

In order to reduce the influence of oscillations on the convergence performance of the algorithm in the information updating process, a damping factor λ was introduced when updating the support matrix and the availability matrix, so the updated formula of the support matrix and the availability matrix is as shown in Equation (12):

(12) $$r_{te}(i,k)=\lambda r_{te-1}(i,k)+(1-\lambda )\times r_{tb}(i,k)$$

(4) Update the availability matrix ***W***. The availability $w_{tb}(i,k)$ refers to how appropriate it would be for the data point *k* as the cluster center of the data point *i*. It can be calculated by Equation (13), and the updated formula after introducing the damping factor is shown in Equation (14):

(13) $$w_{tb}(i,k)=\left\{\begin{array}{c} \min\left\{0,r(k,k)+\sum_{i’\neq i,k}\max\{0,r(j,k)\}\right\},i\neq k\\ \sum_{j\neq k}\max\{0,r(j,k)\},i=k \end{array}\right.$$

(14) $$w_{te}(i,k)=\lambda w_{te-1}(i,k)+(1-\lambda )\times w_{tb}(i,k)$$

(5) When the results of the last two iterations are basically unchanged or reach the set maximum number of iterations, stop the iteration and enter the next step; otherwise, repeat step (3) and step (4).

(6) Calculate the decision matrix ***E***. The decision matrix is the sum of the support matrix ***R*** and the availability matrix ***W***. The positive points on the diagonal of the decision matrix ***E*** are the cluster centers, and the number of positive points is the number of source signals.

After the initial cluster centers and the number of source signals are obtained using the AP clustering algorithm, the *K*-means algorithm can be used to update the cluster centers, and finally the mixing matrix can be calculated. Suppose the cluster center is $C=\{c_{1},c_{2},\ldots ,c_{N}\}$, and *N* is the number of source signals calculated by the AP clustering algorithm, then the formula for calculating the Euclidean distance in the *K*-means clustering algorithm is shown in Equation (15):

(15) $$d(x_{i},c_{j})=\sqrt{{\left(x_{i}-c_{j}\right)}^{T}(x_{i}-c_{j})}$$

Using Equation (15), the data points are divided into *N* classes according to the distance between each data point and the cluster center, and the data point set in each class can be expressed as $DS_{n}(n=1,2,\ldots N)$. Then, use the mean value of the data point set to update the cluster center $c_{n}$ according to Equation (16), as follows:

(16) $$c_{n}=\frac{1}{\left|DS_{n}\right|}\sum_{ds\in DS_{n}}ds$$

After each update of $c_{n}$, use Equation (17) to calculate the objective function of the K-means algorithm, that is, the sum of squares of errors of the classification.

(17) $$E=\sum_{i=1}^{N}\sum_{x_{j}\in S_{i}}{\left(x_{j}-c_{i}\right)}^{2}$$

The K-means algorithm calculates the minimum value of the objective function by continuously iterating Equations (16) and (17) so as to continuously update the cluster centers and finally obtain the optimal cluster center of each set. The matrix formed by each cluster center after the final iteration is the calculated signal mixing matrix. Because that mixing matrix is formed by random arrangement, the output sequence of each reconstruction signal cannot be guaranteed to be consistent with the input sequence of the source signal when the subsequent source signal is recovered.

#### 2.5.3. Damping Coefficient Adaptive Method

Different values of damping coefficient λ will affect the global and local search ability of the algorithm and then interfere with the convergence performance of the algorithm. In the traditional AP algorithm for clustering, the damping coefficient is often set to a fixed value based on prior experience, which makes the algorithm unable to dynamically adjust the search performance at different stages. In view of this, we proposed a dynamic damping coefficient adaptive method. A sliding window with the length of *l* was adopted to compare whether the number of the clusters in the current iteration is reduced or consistent with the number of the clusters in the previous iteration, if so, the number is marked as 1, otherwise, the number is marked as 0. Considering the instability in the initial stage of the algorithm and the occasional small number of oscillations, it can be regarded that when more than 2/3 of the records show 0, oscillations occur. In this time, the damping coefficient λ is adjusted. Considering the convergence of the algorithm, the initial value of λ is set to the system default value of 0.5, and it will not increase when it reaches the maximum value. The specific adjustment rule is shown in Equation (18):

(18) $$\lambda =\lambda_{old}+0.01\;\;\;\lambda \in [0.5,1]$$

where $\lambda_{old}$ denotes the damping coefficient value used in the last iteration.

#### 2.5.4. Cluster Evaluation Indicators

The Silhouette Coefficient (SC), as a measure of clustering results, reflects the difference between the similarity of a sample and other samples in the same cluster and the similarity of samples in different clusters. The closer the Silhouette Coefficient is to 1, the better the clustering result is; the closer the Silhouette coefficient is to −1, the worse the clustering result is. In this study, we used the Silhouette Coefficient to evaluate the clustering results. It can be calculated using Equation (19), as follows:

(19) $$SC(i)=\frac{z(i)-h(i)}{\max\{h(i),z(i)\}}$$

where *h*(*i*) is the average distance between vector *i* and all other samples in the class; and *z*(*i*) is the minimum of the average distance from vector *i* to the samples in each of the other classes.

### 2.6. Source Signal Reconstruction Based on l_1_ Norm

In the underdetermined case, the estimated mixing matrix is a non-full rank matrix, and the source signal cannot be reconstructed by the non-full rank matrix. Therefore, we used *l*_1_ norm [26] to reconstruct the source signal. For the transient linear mixed model of Equation (1), under the condition that the mixing matrix ***A*** has been estimated, the estimation problem of the sparse source signal *S* can be transformed into the following optimization problem, as shown in Equation (20):

(20) $$\hat{s}(t)=\underset{s(t)}{\min}\sum_{i=1}^{N}\left|s_{i}(t)\right|,s.t.:As(t)=x(t),t=1,\cdots ,T$$

where $\hat{s}(t)$ denotes the estimation of source signal s(t).

Since the number of source signals and the number of observed signals are *N* and *M*, respectively, there are at least *N* − *M* zeros in the minimum *l*_1_ norm solution. In other words, there are at most *M* non-zero values, and $C_{N}^{M}$ possible solutions can be obtained [27,28]. Through comparing these possible solutions, the *l*_1_ norm minimization solution can be obtained, and the specific steps of the algorithm are as follows:

(1) Calculate $C_{N}^{M}$ M×M dimensional submatrices of the mixing matrix ***A***, set:

(21) $$B_{k}=[a_{k_{1}},\ldots ,a_{k_{m}}]$$

where $k=1,\cdots ,C_{N}^{M};k_{m}\in \{1,\cdots ,N\}$

(2) For a certain time *t*, solve the possible solutions of the *l*_1_ norm minimization problem, mark as follows:
  (22) $${\hat{s}}^{\left(k\right)}(t)={\left[{\hat{s}}_{1}^{\left(k\right)}(t),\ldots ,{\hat{s}}_{N}^{\left(k\right)}(t)\right]}^{T},k=1,\ldots ,C_{N}^{M}$$
  (23) $$\left\{\begin{array}{ll} {\left[{\hat{s}}_{k_{1}}^{\left(k\right)}(t),\ldots ,{\hat{s}}_{k_{M}}^{\left(k\right)}(t)\right]}^{T}= & B_{k}^{-1}x(t),k_{1},\ldots ,k_{M}\in \left\{1,\ldots ,N\right\}\\ {\hat{s}}_{j}^{\left(k\right)}(t)=0, & j\in \left\{1,\ldots ,N\right\}\mathrm{and}j\neq k_{1},\ldots ,k_{M} \end{array}\right.$$
(3) According to Equation (24), calculate *l*_1_ norm $J_{k}$$,k=1,\ldots ,C_{N}^{M}$ corresponding to ${\hat{s}}^{\left(k\right)}(t)$:
  (24) $$J_{k}=\sum_{i=1}^{N}\left|{{\hat{s}}_{i}}^{\left(k\right)}(t)\right|,k=1,2,\ldots ,C_{N}^{M}$$
(4) According to Equation (25), determine the minimum *l*_1_ norm solution ${\hat{s}}_{\min}(t)$, and take it as the estimation of s(t):
  (25) $$\hat{s}(t)={\hat{s}}_{\min}(t)=\underset{{\hat{s}}^{\left(k\right)}(t)}{\min}J_{k},k=1,2,\ldots ,C_{N}^{M}$$
(5) Repeat steps (2)–(4), until the $\hat{s}(t)$ at all moments is calculated, to obtain the estimation of the source signal.

### 2.7. Measurement Indicators

In order to measure the quality of the reconstructed audio, we introduced the Similarity Coefficient, signal-to-noise ratio, and mean square error.

The Similarity Coefficient $\xi_{ij}$ is a measure of the BSS performance with the Similarity Coefficient of the separated output signal ${\hat{s}}_{i}(t)$ to the source signal $s_{i}(t)$. It can be calculated using Equation (26), as follows:

(26) $$\xi_{ij}=\xi ({\hat{s}}_{i}(t),s_{i}(t))=\frac{\left|\sum_{t=1}^{N}{\hat{s}}_{i}(t)s_{i}(t)\right|}{\sqrt{\sum_{t=1}^{N}{{\hat{s}}_{i}}^{2}(t)\sum_{t=1}^{N}{s_{i}}^{2}(t)}}$$

where $s_{i}(t)$ denotes the *i*-th source signal and ${\hat{s}}_{i}(t)$ denotes the *i*-th estimated source signal. The value range of $\xi_{ij}$ is [0,1]. When $\xi_{ij}$ = 1, it indicates that the *i*-th separated signal and the *i*-th source signal have exactly the same waveform. When $\xi_{ij}$ = 0, ${\hat{s}}_{i}(t)$ and $s_{i}(t)$ are independent of each other. The larger the value of $\xi_{ij}$, the more similar the two are.

The average recovery signal-to-noise ratio (ARSNR) was employed as the evaluation criterion of the source signal recovery performance to describe the distortion degree of the reconstructed signal compared with the source signal. It can be calculated by Equation (27), as follows:

(27) $$ARSNR=\frac{1}{N}\sum_{i=1}^{N}10\mathrm{lg}\frac{E\left[|s_{i}(t){|}^{2}\right]}{E\left[|s_{i}(t)-{\hat{s}}_{i}(t){|}^{2}\right]}$$

where $s_{i}(t)$ denotes the *i*-th source signal and ${\hat{s}}_{i}(t)$ denotes the *i*-th estimated recovered signal. A higher value of ARSNR indicates better recovery performance.

The normalized mean square error (NMSE) is the mean value of the sum of the squares of the errors of the corresponding points of the predicted data and the original data, and it can be calculated by Equation (28):

(28) $$NMSE=10\mathrm{lg}\left(\frac{\sum_{i=1}^{N}(s_{i}-{\hat{s}}_{i}{)}^{2}}{\sum_{i=1}^{N}(s_{i}{)}^{2}}\right)$$

The value of *NMSE* indicates the difference between the source signal and the reconstructed signal, and the smaller the value is, the better the effect is.

## 3. Results and Analysis

According to the proposed algorithm, we intended to solve the problem of underdetermined pig blind source separation in the MATLAB simulation environment. The monophonic audio of oinks, grunts, growls, eating sounds, and screams of adult Landrace pigs after pretreatment was used as the research object. We recorded audio from three adult Landrace sows from the farm and intercepted valid audio segments from them, dividing these valid audio segments into the five categories of pig audio described above. Each audio segment contained the voice of only one pig, and no other voices were mixed in. From these, five different categories of audio segments with better audio quality were selected for subsequent experiments.

This study used pig audio signals in different states, with signal durations ranging from 12 s to 16 s, for subsequent experiments. The method of zero padding was used to align the audio of different durations, and the artificial amplitude attenuation matrix was used to obtain the observed signal so as to complete the experiment of underdetermined pig blind source separation.

In Section 3.1, first, we selected three pig audio signals, the oink sound, the grunt sound, and the growl sound. Twelve s of audio signals were intercepted for underdetermined blind source separation experiments in the case of three source signals and two observation signals to verify the overall feasibility of the algorithm. Then all combinations of three out of five audios, for a total of $C_{5}^{3}$ combinations, were selected and the average value in the 3 × 2 case was computed, thus preventing chance and ensuring the generalization of the algorithm’s performance demonstration. We explored the effects of audio duration and different mixing matrices on the performance of underdetermined blind source separation algorithms. In Section 3.2, different numbers of source signals, observed signals, and amplitude attenuation matrices were set, and then the reconstruction ability of the algorithm was compared with other algorithms in the literature, and the performance of the algorithm was verified using the measurement indicators in Section 2.7.

### 3.1. UBSS for Three Source Signals and Two Observed Signals

**Figure 2:**
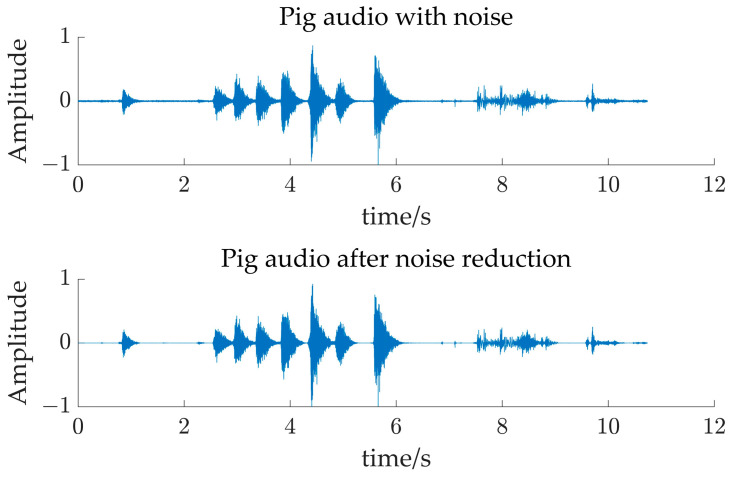
Comparison of pig audio before and after noise reduction.

Prior to the UBSS experiments, the recorded audio needed to be noise-canceled. Although a quiet environment was selected to record the audio of the pig alone to exclude most of the external environmental noise, there is still the bottom noise generated by the recording equipment, so this study uses the Kalman filter noise reduction algorithm to reduce the noise. Figure 2 demonstrates the audio time-domain comparison plot before and after Kalman filtering, which clearly shows that most of the bottom noise of the audio is removed, reducing the subsequent impact on the underdetermined blind source separation experiments.

**Figure 3:**
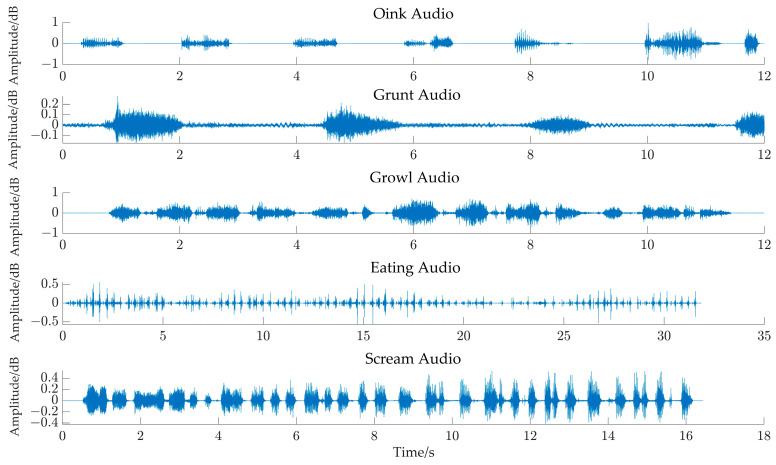
Original audio signal waveform of pigs in different states.

**Figure 4:**
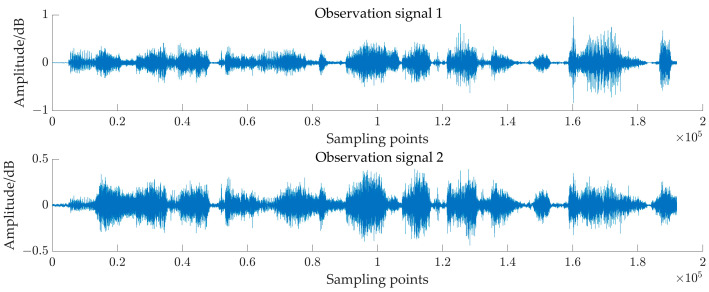
Waveform of pig observed audio signal.

Taking the case of three source signals and two observed signals as an example, we tested the UBSS in this study. Using the amplitude attenuation matrix Equation (29) to superimpose the three source signals of pig oinks, grunts, and growls in Figure 3, we obtained the waveform of the observed audio signal, as shown in Figure 4.

**Figure 5:**
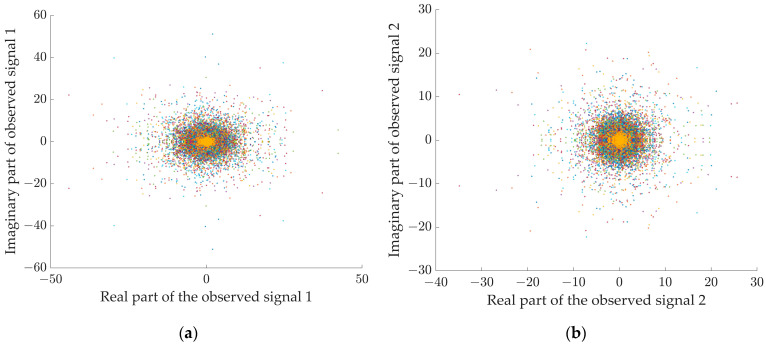
Scatter plots of observed signals with a duration of 12 s in the time-frequency domain. (**a**) Observed signal 1, (**b**) observed signal 2.

Then, taking 44.1 kHz as the uniform sampling rate, we obtained the observed audio signal of 12 s, as shown in Figure 5. After that, short-time Fourier transform was performed on the observed signals, a Hanning window was selected as a window function, the window size was set to 512, the window overlap was 256, and the complex matrices of the two observed signals were finally obtained.

(29) $$A=\left[\begin{array}{ccc} 0.9552 & 0.3663 & 0.6205\\ 0.2960 & 0.9974 & 0.5550 \end{array}\right]$$

**Figure 6:**
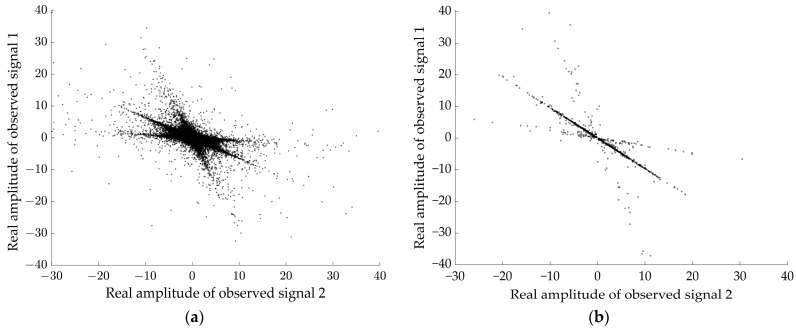
Scatter plots of the real part of observed signal and extracted single source points with a duration of 12 s in the time-frequency domain. (**a**) Observed signal, (**b**) single source points.

Figure 6a gives the scatter plot of the complex matrix visualization after STFT of the observed signals. According to the method proposed in Section 2.3, set M=6, $\varepsilon_{1}=0.01$, $\varepsilon_{2}=0.05$, σ=0.5 to extract the single source point. Figure 6b shows that the amplitude of the signal is clearly distributed in three straight lines on the 2D plane after the single source point screening by the method proposed in this study, and the low-energy points have been basically eliminated.

In this study, we applied an improved AP clustering algorithm to cluster the extracted feature single source points. During the experiment, the maximum number of iterations was set to 500, the number of iteration termination was set to 50, the damping coefficient was adjusted using the adaptive rules, the window length *l* was set to 6, the initial damping coefficient λ was set to 0.5, and the final number of clusters before each damping coefficient adjustment was recorded.

**Figure 7:**
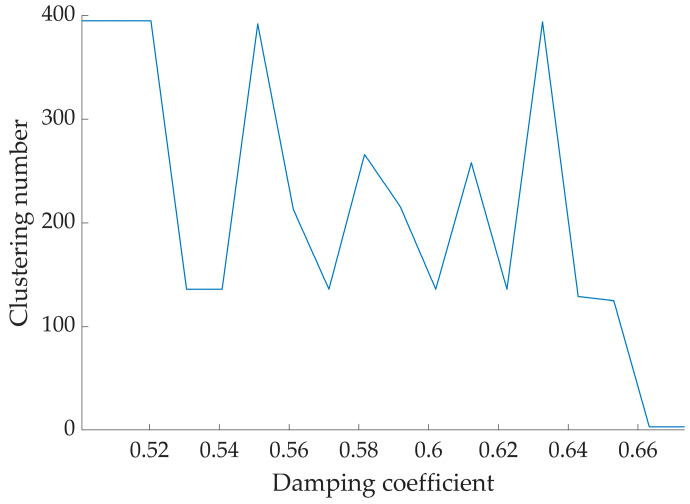
Different damping coefficients and clustering results.

**Figure 8:**
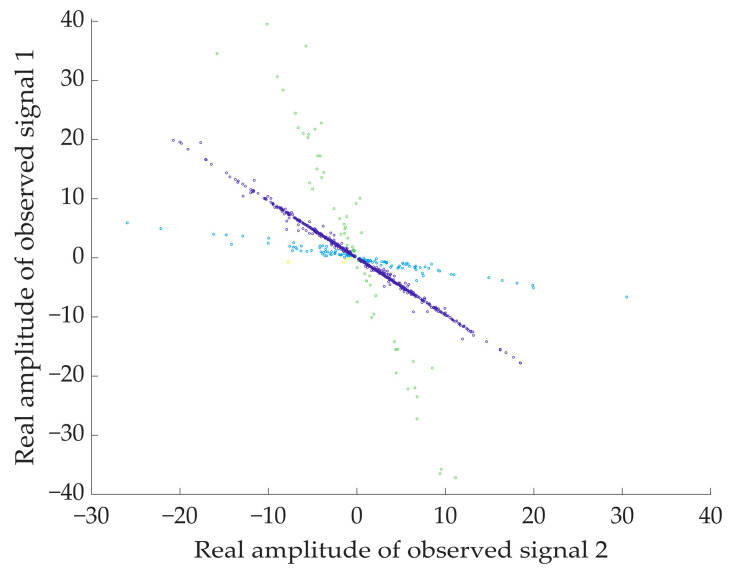
Clustering results of improved AP.

Figure 7 shows the damping coefficients during clustering and the variation curve of the clustering results. With the gradual increase of the number of iterations, when λ is at the initial value 0.5, the clustering results are larger and the values oscillate. With the increase of the damping coefficient, the number of clusters is also changing, and when the value of λ increases to 0.67, the number of clusters tends to be stable. Figure 8 presents the real part clustering results of the improved AP algorithm for the observed signals. It can be clearly seen that the feature points in a straight line are clustered into one class, and there are three classes in total, which are represented by different colors.

**Figure 9:**
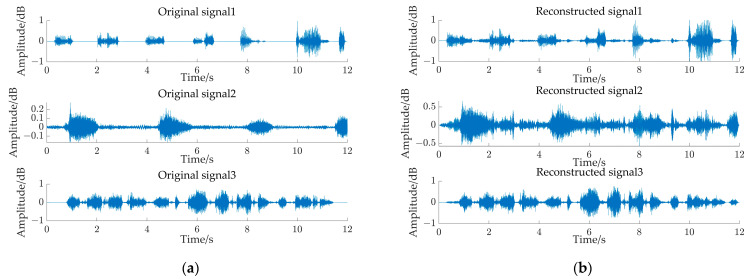
Waveforms of source and reconstructed pig audio signals for 12 s pig observed signals. (**a**) Source audio signal, (**b**) reconstructed audio signal.

Figure 9 shows the waveforms of source and reconstructed pig audio signals for 12 s observed signals. The arrangement order of the reconstructed audio signals is not consistent with the input order of the source signals. The authors of reference [29] used clustering by frequency to solve the ranking ambiguity problem. However, this study focused on the blind source separation of underdetermined pig audio signals by the “two-step method”, so we did not discuss the ranking problem here. From the waveform, the source signals 2 and 3 are roughly the same as the corresponding reconstructed signals, with a slight difference in amplitude. The difference mainly lies in the invalid audio (noise) segment, and the source signal 1 is significantly different from the corresponding reconstructed signal. Comparing the source signal with the reconstructed signal, it can be known that the reconstructed signal adds many other bands on the basis of the source signal waveform, possibly because the source signal 1 has more mute segments, and after mixing with other audio signals, the characteristics of each mute segment are no longer obvious, which is greatly affected by other source signals, making the final results different.

In order to examine the effect of different time lengths of audio signals on the reconstructed audio quality, Table 1 lists the average Similarity Coefficients, ARSNR, and NMSE for all combinations in the 3 × 2 case. It can be seen that the audio duration has a certain effect on the audio reconstruction effect, the longer the duration, the better the reconstruction effect. From the data in Table 1, it can be seen that the enhancement from 5 s to 9 s is greater than that from 9 s to 12 s, indicating that the reconstruction effect does not increase linearly with the audio duration, but rather the reconstruction effect will not change much after a certain duration.

In order to investigate the effect of different mixing matrices on the audio reconstruction effect, the mixing matrices are constructed using the following equations. Table 2 lists the average Similarity Coefficient, ARSNR, and NMSE values for all combinations in the 3 × 2 case, with the audio duration selected as 9 s. From the data in Table 2, it can be seen that different mixing matrices have an effect on the reconstruction effect. The mixing matrix represents the degree of attenuation of each source signal to the recording equipment, which is greatly related to the placement of the recording equipment. The farther away the recording equipment is from the source, the more serious the attenuation of the signal is, which also greatly affects the reconstruction of the signal.

(30) $$A=\left[\begin{array}{ccc} \cos(T{}^{\circ}) & \cos(2T{}^{\circ}) & \cos(3T{}^{\circ})\\ \sin(T{}^{\circ}) & \sin(2T{}^{\circ}) & \sin(3T{}^{\circ}) \end{array}\right]$$

### 3.2. Reconstruction Analysis of Multi-Channel Source Signals and Multi-Channel Observed Signals

**Figure 10:**
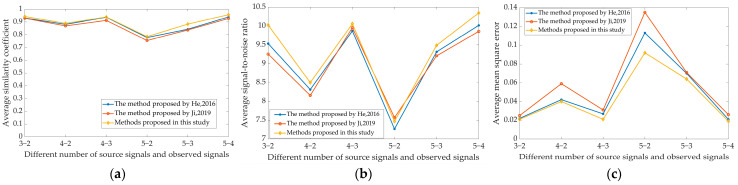
Reconstruction indicators under different number of source signals and observed signals. (**a**) Average similarity coefficient, (**b**) average signal-to-noise ratio, (**c**) average mean square error. He’s method corresponds to the reference [30]. Ji’s method corresponds to the reference [31].

In order to evaluate the algorithm performance, we selected five kinds of pig audio signals, including oinks, grunts, growls, eating sounds, and screams, and set 3×2, 4×2, 4×3, 5×2, 5×3, and 5×4 attenuation matrixes with different amplitudes to construct different numbers of observed signals that are less than the number of source signals. Then, we used the underdetermined blind source separation algorithm to separate the observed signals and made a comparison with the methods in references [30,31]. The comparison values of the measurement indicators are shown in Figure 10, in which the coordinate number of the *x*-axis is “the number of source signals—the number of observed signals”, and the value displayed on the *y*-axis is the average value of the corresponding evaluation indicators measured by all the separated signals and the source signals.

As shown in Figure 10, the separated audio quality indicators are different for different numbers of source signals and observed signals. When the number of source signals is constant, the more the number of observed signals is, the better the quality indicators measured by each method are and the more reliable the separated audio is; for the Similarity Coefficient, the values in references [30,31] are 0.778–0.939 and 0.755–0.927, respectively, while that measured by the method proposed in this study is 0.785–0.957; in the aspect of signal-to-noise ratio, the values of reference [30,31] are 7.268–10.017 dB and 7.568–9.897 dB, respectively, while that measured by the method proposed in this study is 7.468–10.347 dB; for the average mean square error, the values of references [30,31] are 0.021–0.113 and 0.025–0.135, respectively, while that measured by the method proposed in this study is 0.019–0.092; on the whole, the method proposed in this study has higher values in average Similarity Coefficient and average signal-to-noise ratio, and lower values in average mean square error, so it is better than the methods described in references [30,31].

## 4. Discussion

Because it is difficult to separate the pig audio aliasing signal collected in the group environment of pig farms, how to improve the performance of blind source separation has become a challenging and practical topic in signal processing for healthy pig breeding. In view of this, this study proposes an underdetermined pig blind source separation method based on sparsification theory. We took audio signals obtained by mixing the audio signals of pigs in different states according to different coefficients as the observed signals and used short-time Fourier transform (STFT) to carry out the time-frequency domain conversion of the audio signals. Then, the single source points in the signals were screened by grouping, and the single source points were clustered using the adaptive damping coefficient AP algorithm combined with singular value decomposition to estimate the mixing matrix. Finally, the *l*_1_ norm method was employed to reconstruct the pig audio signals.

The experimental results show that both the audio duration and the mixing matrix have a certain effect on the underdetermined blind source separation algorithm, so the recording duration and the spatial location of the recording equipment must be taken into account in the practical application of the blind source separation algorithm. The simulation results show that the proposed algorithm is better than other classical BSS algorithms in estimating the mixing matrix and separating the signals in synthetic data sets, and it also improves the estimation accuracy of the source signals.

This study is only in an ideal experimental environment and does not fully take into account various factors in the real pigsty environment, such as the influence of a large number of external ambient noises and the reverberation generated by the impulse response of the room. In a real swine barn environment, a large amount of external ambient noise, such as wind, mechanical noise, and other animal sounds, may have a significant effect on the separation results. These noise sources can increase the complexity of the mixed signal and pose a greater challenge to blind source separation algorithms.

Another important influence is the reverberation effect caused by Room Impulse Response (RIR). In closed or semi-enclosed environments, reflections of sound off walls, floors, and other surfaces cause reverberation, which not only blurs the source signa, but also makes estimation of the mixing matrix more difficult. Reverberation effects usually require complex pre-processing and post-processing steps, such as the use of de-reverberation algorithms to minimize their effects.

In summary, although this study achieved some results in an ideal experimental environment, in practical applications, various factors such as ambient noise, reverberation effects, equipment characteristics, and their arrangement need to be considered to optimize the performance of blind source separation algorithms. Future research should further explore the effects of these factors and develop more robust and efficient algorithms to adapt to complex and changing practical environments.

## Figures and Tables

**Table 1:** Impact of different audio durations on reconstruction performance.

Duration of Audio	Similarity Coefficient	ARSNR/dB	NMSE
5 s	0.812	8.685	0.016
9 s	0.847	9.064	0.008
12 s	0.854	9.152	0.007

**Table 2:** Effect of different mixing matrices on reconstruction performance.

Value of T	Similarity Coefficient	ARSNR/dB	NMSE
15	0.829	8.797	0.012
25	0.877	9.386	0.005
35	0.838	9.023	0.009

## Data Availability

Data are contained within the article.
